# Shifting risk profiles in the systemic-to-focal transition of brucellosis: a multicenter analysis of spondyloarthritis development

**DOI:** 10.1186/s12879-026-13685-w

**Published:** 2026-06-02

**Authors:** Meng Zhao, Xuefeng Luo, Abudusalamu Alimujiang, Junru Liao, Junchao Ma, Jian Guo, Xi Wang, Jun Zhang, Ziyi Xu, Qiyu Jia, Chuang Ma

**Affiliations:** 1 Department of Trauma Orthopedics, The First Affiliated Hospital of Xinjiang Medical University, Urumqi, Xinjiang China; 2 Department of Reconstructive Microsurgery, The Karamay Central Hospital, Karamay, Xinjiang China; 3 School of Medicine, Nankai University, Tianjin, China

**Keywords:** Brucellosis, Osteoarthritis, Clinical characteristics, Risk factors

## Abstract

**Background:**

Brucellar Spondyloarthritis (BrSpA), a common and disabling complication of brucellosis, often develops as the disease transitions from its acute systemic phase. However, the specific risk factors and clinical patterns heralding this shift are poorly understood. This study aimed to identify stage-dependent risk factors for BrSpA and to elucidate the paradoxical clinical signs associated with its development.

**Methods:**

In this retrospective, multicenter study, we analyzed data from 642 participants, comprising a BrSpA group (*n* = 187), a non-osteoarticular brucellosis group (*n* = 176), and a healthy control group (*n* = 279). Baseline data, clinical symptoms and laboratory findings were collected, summarized and analyzed. The significance of the data was determined using t-test or Pearson chi-square test, and logistic regression test was used to calculate the odds ratio (OR) to characterize the relevant risk factors affecting the development of BrSpA.

**Results:**

When compared to the non-osteoarticular brucellosis group, progression to the subacute/chronic stages, lower educational attainment, fatigue, and musculoskeletal pain were independent risk factors for BrSpA. In this same comparison, factors reflecting acute systemic inflammation (e.g., anorexia, elevated C-reactive protein [CRP]) were paradoxically associated with a lower risk of BrSpA (OR < 1). In contrast, when the BrSpA group was compared to healthy controls, these same factors were confirmed as significant risk factors (OR > 1).

**Conclusion:**

The development of BrSpA in brucellosis is marked by a paradoxical clinical profile. Our findings demonstrate that the resolution of systemic inflammatory signs should not be uniformly interpreted as recovery. Instead, this pattern may represent a critical transition to focal disease, serving as a key warning sign for clinicians to heighten vigilance and initiate proactive diagnosis to prevent long-term complications.

## Introduction

Brucellosis, a zoonotic disease caused by Brucella species, poses a major global health threat, spreading via macrophages to multiple organs and potentially causing multi-systemic complications [1, 2]. Osteoarticular involvement, such as spondylitis, peripheral arthritis, or sacroiliitis, is a common and debilitating sequelae, especially in mid-to-late disease stages [3–7]. With an estimated 1.6–2.1 million new cases annually worldwide, brucellosis incidence is alarmingly on the rise [8], and its true global burden is likely underestimated, despite being recognized by the Food and Agriculture Organization of the United Nations (FAO) and the World Health Organization (WHO) as a widespread zoonosis [9–11]. It also poses a lab infection risk and causes socioeconomic burdens [12–14].

A critical challenge in managing brucellosis is the non-specific early symptoms, often leading to delayed diagnosis [15]. The aforementioned osteoarticular manifestations (spondylitis, peripheral arthritis, sacroiliitis), when caused by Brucella, share core features with the broader rheumatological category of Spondyloarthritis (SpA). SpA encompasses a group of systemic inflammatory diseases primarily affecting the axial skeleton, peripheral joints, and entheses. Thus, for the purpose of this study and to reflect its inflammatory nature and specific clinical pattern, we will refer to this constellation of brucellar osteoarticular complications as Brucellar Spondyloarthritis (BrSpA). It’s important to note the distinctions between SpA and BrSpA. SpA is a chronic inflammatory disease marked by inflammatory low back pain, restricted spinal mobility, and peripheral arthritis, treated mainly with NSAIDs for anti-inflammatory and analgesic effects. BrSpA, an infectious spondyloarthritis from Brucella infection in mid-to-late brucellosis, causes joint deformities, chronic pain, disability, and psychological trauma, and is treated primarily with antibiotics [16, 17]. Despite its prevalence and impact, the transition from uncomplicated brucellosis to BrSpA is not fully understood. Specifically, how clinical and laboratory features evolve during this progression, and what specific factors predispose patients to develop these spondyloarthritis-like manifestations, remain inadequately elucidated. Current clinical studies are limited, hampering the development of targeted preventive and therapeutic strategies for BrSpA. Interestingly, as brucellosis evolves from acute to chronic, patients developing BrSpA may show complex changes. For example, an “improvement” in systemic symptoms or inflammatory markers might paradoxically signal a higher risk of BrSpA. Understanding these shifts is vital for risk assessment.

This study, therefore, aims not only to identify conventional risk factors for BrSpA but also to meticulously explore these evolving characteristics in patients progressing through different disease phases. Through a retrospective analysis of 642 cases, comparing patients with established BrSpA, brucellosis without such osteoarticular complications, and healthy controls, we seek to uncover the nuanced patterns and specific risk profiles associated with the development of BrSpA. By elucidating these factors, we intend to provide a more refined framework for early risk stratification, facilitate timely and targeted clinical management, and ultimately improve outcomes for patients with brucellosis at risk of developing these severe osteoarticular complications.

## Materials and methods

### Selection of study participants

A total of 642 cases were enrolled in this study, including 314 first-diagnosed brucellosis patients hospitalized in the First Affiliated Hospital of Xinjiang Medical University (Center I) from January 2014 to December 2023 and 237 healthy people with outpatient physical examination versus 49 first-diagnosed cases hospitalized in Karamay Central Hospital (Center II) from January 2019 to December 2023 brucellosis patients and 42 healthy people with outpatient physical examination. And according to the results of imaging examinations, after excluding other causes of osteoarthritic injuries, all patients were divided into three groups: BrSpA group (187 cases), brucellosis non-osteoarthritis group (176 cases), and healthy control group (279 cases). The risk factors associated with BrSpA were determined by comparing the baseline data, clinical symptoms and laboratory examination characteristics of the three groups. The Ethics Committee of The First Affiliated Hospital of Xinjiang Medical University granted an informed consent exemption for the study (12.12.2024/K202412–36). Patients’ personal information was anonymized and de - identified prior to analysis. During the study phase, three blinding strategies were employed: (1) Data collectors were blinded to case/control status by using de-identified forms with study IDs only; (2) Subjective assessments (e.g., imaging features) were independently evaluated by experts unaware of group allocation; 2. Initial statistical analyses were conducted using blinded study IDs, with group labels revealed only after results were finalized.

### Data collection

Electronic medical records of all enrolled patients were retrospectively analyzed, and clinical data were extracted. The data included the following: gender, age, education level, area of residence, smoking history, alcohol consumption history, BMI, and whether there was any combination of hypertension and diabetes mellitus. Staging: acute stage (0–3 months), subacute stage (3–6 months), chronic stage (> 6 months). Whether symptoms of fever, hyperhidrosis, fatigue, anorexia, enlarged lymph nodes, splenomegaly, white blood cells count, lymphocytes count, neutrophils count, red blood cell count, hemoglobin, platelets count, erythrocyte sedimentation rate (ESR), C-reactive protein(CRP), lactate dehydrogenase (LDH), aspartate aminotransferase (AST), alanine aminotransferase (ALT), and other laboratory indices were present.

### Inclusion criteria

The diagnosis of brucellosis is based on the “Diagnostic Criteria for Brucellosis (WS269-2019)” and the “Brucellosis Diagnosis and Treatment Protocol (2023 Edition)” issued by the General Office of the National Health Commission of China in 2023. It requires a combination of relevant epidemiological history (for instance, a close contact history between the patient and suspected brucellosis-infected livestock or livestock products prior to the onset of the disease, or a history of consuming raw cow or sheep milk and meat products, or residing in a brucellosis-endemic area, or engaging in work such as brucella culture, detection, or the production and use of brucella vaccines), a positive Rose Bengal Plate Test (RBPT) with a Standard Tube Agglutination Test (SAT) titer of 1:100 or higher, or the isolation of Brucella from blood culture.

On this basis, in patients with brucellosis, a diagnosis of BrSpA requires both clinical features (joint swelling, back pain, and restricted movement) and positive imaging findings. Clinical features must be assessed by at least two independent physicians, and imaging findings by at least two experienced radiologists. Imaging findings supporting a diagnosis of BrSpA are defined as follows:

X-ray: narrowing of the joint space and irregular, rough articular surfaces; Computed Tomography (CT): worm-eaten (moth-eaten) bone erosion or vertebral body destruction, with osteophytes and bony bridges surrounding the lesions; Magnetic Resonance Imaging (MRI) and/or bone scans: bone marrow edema, worm-eaten or patchy irregular destructive lesions, and paravertebral abscesses [18–21].

**Fig. 1:**
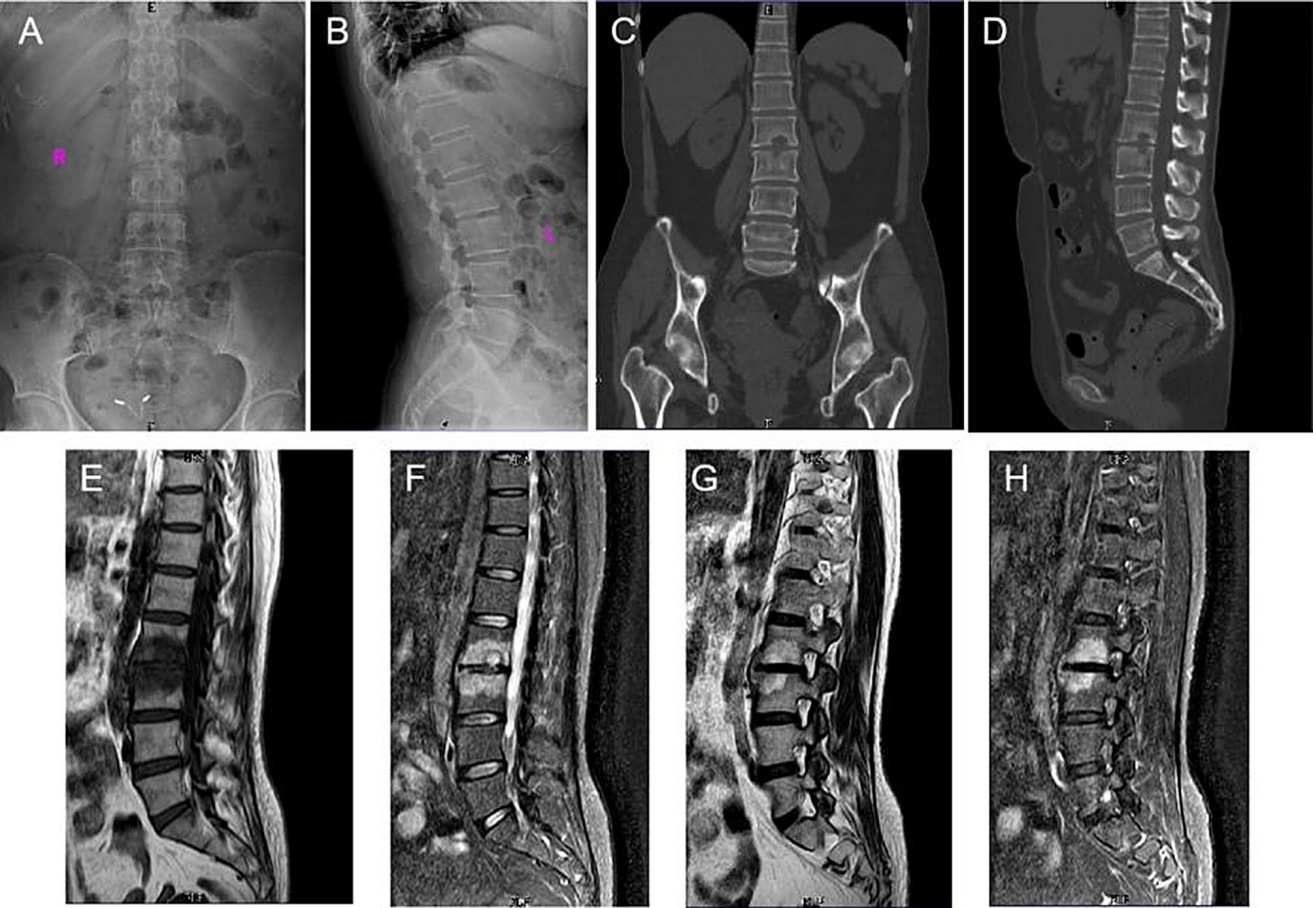
Imaging images of typical cases of brucellar spondylitis. Brucellar spondylitis. **A**-**B**: X-ray films show the intervertebral space at L2-L3 is slightly narrowed. **C**-**D**: CT images show the intervertebral space between the L2 and L3 vertebral bodies is slightly narrowed, a round-like hypodense shadow is visible within the vertebral bodies, with slightly increased bone density in the surrounding area. The anterior margin of the L2 vertebral body exhibits an irregular morphology, characterized by osteophyte formation and partial discontinuity of the bone cortex. **E**-**H**: MRI images show the intervertebral space between the L2 and L3 vertebral bodies is slightly narrowed. Patchy areas of long T1 and slightly long T2 signal intensity are observed within the bone substance at the superior and inferior margins of the L2 and L3 vertebral bodies, which appear hyperintense on the fat-suppression sequence. The L2-L3 intervertebral disc demonstrates heterogeneous signal intensity, with localized slightly hyperintense regions on the fat-suppression sequence.

**Fig. 2:**
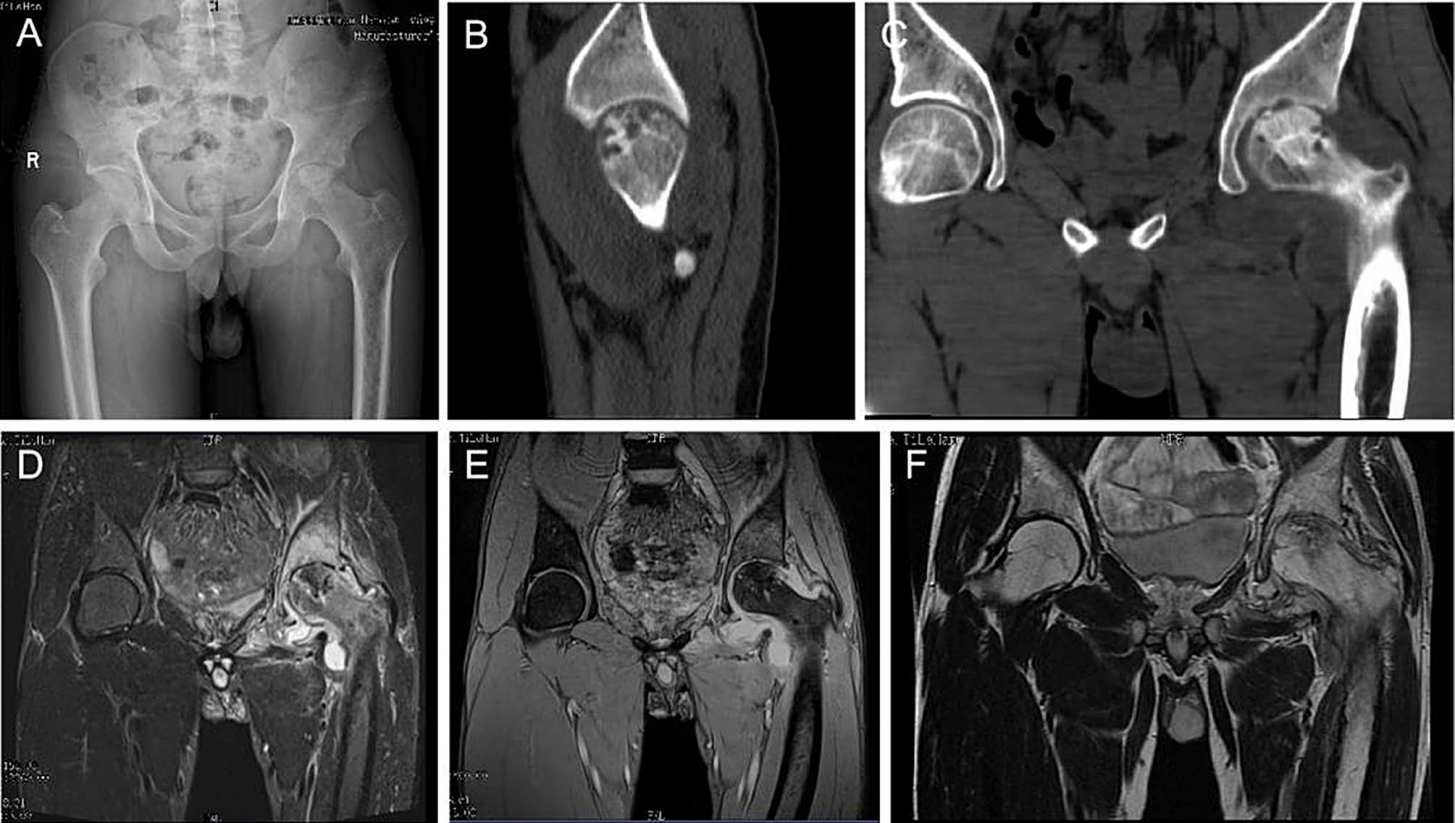
Imaging images of typical cases of brucellar peripheral arthritis. Brucellar peripheral arthritis. **A**-**C**: CT images show abnormal bone lesions are present in the left acetabulum, left femoral head, and femoral neck, with hypodense regions observed in the adjacent muscular compartments. **D**-**F**: MRI images show destructive bone lesions are present in the left femoral head. Marrow edema is observed in the left femoral head, femoral neck, greater trochanter, proximal femoral shaft, left acetabulum, and left ischium, accompanied by the formation of a surrounding abscess

Joint fluid analysis and bone tissue biopsy are of great significance for the etiological diagnosis of BrSpA, but they are not routine examinations for all patients. Given their invasive nature and higher risks, they are typically considered only in cases where serological results are negative or ambiguous but with high clinical suspicion, when differentiation from other diseases is required, and when standard antibiotic therapy fails. Typical cases are shown in Figs. 1 and 2.

### Exclusion criteria

(1) patients with incomplete relevant information; (2) patients who are unable to communicate, such as mental abnormality; (3) patients with other osteoarthritic diseases; (4) patients with underlying diseases that predispose to osteoarthritic lesions (e.g., osteoporosis, tuberculosis, tumors, and rheumatism); and (5) patients applying medications that predispose to osteoarthritis (e.g., glucocorticoids).

### Statistical analysis

This study was statistically analyzed using IBM SPSS Statistics 27 (IBM Corp, USA). Age and BMI, as continuous variables, were analyzed by the Wilcoxon rank - sum test and expressed as median and interquartile range [M (Q1, Q3)].

In addition, gender, education level (below high school, high school and above), region of residence (farming and pastoral areas and non - farming and pastoral areas), smoking (yes or no), drinking (yes or no), hypertension (yes or no), diabetes (yes or no), Disease Stage (acute, subacute, chronic), fever (yes or no), hyperhidrosis (yes or no), fatigue (yes or no), anorexia (yes or no), enlarged lymph nodes (yes or no), and splenomegaly (yes or no) were treated as categorical variables.

Based on the reference ranges of laboratory tests in China, the following examination indicators were classified as categorical variables: White blood cell count: >9.5 × 10⁹/L, 3.5–9.5 × 10⁹/L, < 3.5 × 10⁹/L; Lymphocyte count: <1.1 × 10⁹/L, ≥ 1.1 × 10⁹/L; Neutrophil count: <1.8 × 10⁹/L, ≥ 1.8 × 10⁹/L; Red blood cell count: <3.8 × 10¹²/L, ≥ 3.8 × 10¹²/L; Hemoglobin: <110 g/L, ≥ 110 g/L; Platelet count: <100 × 10⁹/L, ≥ 100 × 10⁹/L; ESR: <15 mm/h, ≥ 15 mm/h༛CRP: <10 mg/L, ≥ 10 mg/L; LDH: >250 U/L, ≤ 250 U/L; AST: >40 U/L, ≤ 40 U/L༛ALT: >40 U/L, ≤ 40 U/L.These categorical variables were analyzed numerically using the Pearson χ² test or Fisher exact test. Variables with a *P*-value < 0.05 in the t-test, Pearson χ² test, or Fisher exact test were included in the multifactorial logistic regression analysis, and the odds ratio (OR) and 95% confidence intervals (CI) were calculated. Statistical tests were two-sided, and significance was defined as a *P*-value < 0.05.

## Results

### Population characteristics

Of the 363 patients with brucellosis, the male-to-female sex ratio was 2.3:1.0 (252:111), and the age was 50 (39, 58) years. In the BrSpA and brucellosis non-osteoarthritis groups, the male to female sex ratio was 2.8:1.0 (138:49) and 1.8:1.0 (114:62), respectively, and the age was 51 (41,60) and 49 (39,56) years, respectively. Distribution of affected joints in patients with BrSpA: 137 cases (73.3%) were spondylitis, with lumbar spine being the most affected (118 cases); 51 cases (27.3%) were peripheral arthritis, with the knee being the most affected (23 cases); 19 cases (10.2%) were sacroiliac arthritis, with 11 cases bilaterally and 8 unilaterally. Eighteen patients had simultaneous involvement of 2 or more sites, accounting for 9.6%. Table 1 for details.

**Table 1:** Distribution of affected joints in patients with BrSpA (*n* = 187)

Type	Affected site	Number of cases (*n*)	Percentage (%)
Spondylitis	Cervical spine	12	6.42%
	Thoracic spine	7	3.74%
	Lumbar spine	118	63.10%
Peripheral arthritis	Knee joint	23	12.30%
	Hip joint	15	8.03%
	Shoulder joint	3	1.60%
	Ankle joint	5	2.67%
	Elbow joint	4	2.14%
	Wrist joint	1	0.53%
Sacroiliitis	Unilateral	8	4.28%
	Bilateral	11	5.88%

### Univariate analysis of risk factors: BrSpA vs. non-osteoarticular brucellosis group

The univariate analysis of risk factors showed (Table 2) that the number of patients with BrSpA with below high school level of education, farming and pastoral areas residence, farming and pastoral-related occupations, BMI, subacute stage, and chronic stage patients was significantly higher than the number of patients with brucellosis non-osteoarthritis (all *P* < 0.05). In patients with BrSpA, the incidence of symptoms of fever, hyperhidrosis, and anorexia was lower than that of patients in the brucellosis non-osteoarthritis group (all *P* < 0.05), and the incidence of symptoms of fatigue and muscle and joint pain was higher than that of patients in the brucellosis non-osteoarthritis group (all *P* < 0.05). In patients with brucellosis non-osteoarthritis, the incidence of decreased white blood cell count, lymphocytes count, neutrophils count, red blood cell count, hemoglobin, and platelets count was higher than that of patients in the BrSpA group (all *P* < 0.05), and the incidence of increased CRP, LDH, AST, and ALT was higher than that of patients in the BrSpA group (all *P* < 0.05). There was no statistically significant difference between the two groups in terms of gender, age, history of smoking, history of alcohol consumption, comorbid hypertension, diabetes mellitus, enlarged lymph nodes, splenomegaly, and ESR (all *P* > 0.05).

**Table 2:** Univariate analysis of risk factors: BrSpA vs. non-osteoarticular brucellosis group

Factor	BrSpA group (*n* = 187)	Non-osteoarticular brucellosis group (*n* = 176)	*P*
**Gender**			
Male	138 (73.8%)	114 (64.8%)	0.062
Female	49 (26.2%)	62 (35.2%)	
**Age**			
P50 (P25, P75)	51 (41, 60)	49 (39, 56)	0.094
**Education level**			
Below high school	158 (84.5%)	126 (71.6%)	0.003
High school and above	29 (15.5%)	50 (28.4%)	
**Region**			
Farming and pastoral areas	165 (88.2%)	125 (71.0%)	< 0.001
Non-farming and pastoral areas	22 (11.8%)	51 (29.0%)	
**Occupation**			
Farming and pastoral-related occupations	132 (70.6%)	88 (50.0%)	< 0.001
Other occupations	55 (29.4%)	88 (50.0%)	
**Smoking**			
Yes	56 (29.9%)	45 (25.6%)	0.352
No	131 (70.1%)	131 (74.4%)	
**Drinking**			
Yes	32 (17.1%)	37 (21.0%)	0.343
No	155 (82.9%)	139 (79.0%)	
**BMI**			
P50 (P25, P75)	23.77 (21, 25)	22.81 (20, 24)	0.001
**Hypertension**			
Yes	37 (19.8%)	30 (17.0%)	0.501
No	150 (80.2%)	146 (83.0%)	
**Diabetes**			
Yes	20 (10.7%)	16 (9.1%)	0.609
No	167 (89.3%)	160 (90.9%)	
**Disease stage**			
Acute	39 (20.8%)	111 (63.1%)	< 0.001
Subacute	68 (36.4%)	27 (15.3%)	
Chronic	80 (42.8%)	38 (21.6%)	
**Fever**			
Yes	71 (38.0%)	117 (66.5%)	< 0.001
No	116 (62.0%)	59 (33.5%)	
**Hyperhidrosis**			
Yes	57 (30.5%)	72 (40.9%)	0.038
No	130 (69.5%)	104 (59.1%)	
**Fatigue**			
Yes	95 (50.8%)	68 (38.6%)	0.020
No	92 (49.2%)	108 (61.4%)	
**Anorexia**			
Yes	69 (36.9%)	94 (53.4%)	0.002
No	118 (63.1%)	82 (46.6%)	
**Muscle and joint pain**			
Yes	179 (95.7%)	76 (43.2%)	< 0.001
No	8 (4.3%)	100 (56.8%)	
**Lymphadenopathy**			
Yes	7 (3.7%)	12 (6.8%)	0.189
No	180 (96.3%)	164 (93.2%)	
**Splenomegaly**			
Yes	16 (8.6%)	13 (7.4%)	0.681
No	171 (91.4%)	163 (92.6%)	
**White blood cell count**			
> 9.5 × 10⁹/L	12 (6.4%)	12 (6.8%)	< 0.001
3.5–9.5 × 10⁹/L	174 (93.1%)	128 (72.7%)	
< 3.5 × 10⁹/L	1 (0.5%)	36 (20.5%)	
**Lymphocyte count**			
< 1.1 × 10⁹/L	14 (7.5%)	38 (21.6%)	< 0.001
≥ 1.1 × 10⁹/L	173 (92.5%)	138 (78.4%)	
**Neutrophil count**			
< 1.8 × 10⁹/L	6 (3.2%)	43 (24.4%)	< 0.001
≥ 1.8 × 10⁹/L	181 (96.8%)	133 (75.6%)	
**Red blood cell count**			
< 3.8 × 10¹²/L	15 (8.0%)	46 (26.1%)	< 0.001
≥ 3.8 × 10¹²/L	172 (92.0%)	130 (73.9%)	
**Hemoglobin level**			
< 110 g/L	21 (11.2%)	49 (27.8%)	< 0.001
≥ 110 g/L	166 (88.8%)	127 (72.2%)	
**Platelet count**			
< 100 × 10⁹/L	1 (0.5%)	37 (21.0%)	< 0.001
≥ 100 × 10⁹/L	186 (99.5%)	139 (79.0%)	
**ESR**			
< 15 mm/h	27 (14.4%)	26 (14.8%)	0.928
≥ 15 mm/h	160 (85.6%)	150 (85.2%)	
**CRP**			
< 10 mg/L	43 (23.0%)	26 (14.8%)	0.046
≥ 10 mg/L	144 (77.0%)	150 (85.2%)	
**LDH**			
> 250 U/L	8 (4.3%)	127 (72.2%)	< 0.001
≤ 250 U/L	179 (95.7%)	49 (27.8%)	
**AST**			
> 40 U/L	39 (20.9%)	89 (50.6%)	< 0.001
≤ 40 U/L	148 (79.1%)	87 (49.4%)	
**ALT**			
> 40 U/L	32 (17.1%)	98 (55.7%)	< 0.001
≤ 40 U/L	155 (82.9%)	78 (44.3%)	

### Multifactorial logistic analysis of risk factors: BrSpA vs. non-osteoarticular brucellosis group

The relevant factors that were statistically significant in the univariate analysis were used as independent variables, and the variables with *P* < 0.05 were included in the multifactorial Logistic analysis. The results of the multifactorial analysis showed that (Table 3), below high school education, subacute stage, chronic stage, fatigue, and muscle and joint pain were the independent risk factors for the occurrence of BrSpA in brucellosis (*P* < 0.05), and anorexia, red blood cell count < 3.8 × 10^12^/L, CRP≥10 mg/L, and LDH>250U/L were protective factors for the occurrence of BrSpA(*P* < 0.05).

**Table 3:** Multifactorial logistic analysis of risk factors: BrSpA vs. non-osteoarticular brucellosis group

Factor	B	OR	95%CI	*P*
**Education level**				
High school and above		1.00		
Below high school	1.46	4.32	1.54–12.13	< 0.01
**Disease stage**				
Acute		1.00		
Subacute	2.43	11.35	3.55–36.27	< 0.001
Chronic	1.51	4.52	1.71–11.94	< 0.01
**Fatigue**				
No		1.00		
Yes	3.50	32.94	8.11-133.79	< 0.001
**Anorexia**				
No		1.00		
Yes	-1.50	0.22	0.08–0.66	< 0.01
**Muscle and joint pain**				
No		1.00		
Yes	2.20	9.06	2.95–27.81	< 0.001
**Red blood cell count**				
≥ 3.8 × 10¹²/L		1.00		
< 3.8 × 10¹²/L	-1.69	0.19	0.04–0.89	< 0.05
**CRP**				
< 10 mg/L		1.00		
≥ 10 mg/L	-1.16	0.31	0.11–0.94	< 0.05
**LDH**				
≤ 250 U/L		1.00		
> 250 U/L	-3.79	0.02	0.01–0.07	< 0.001

**Fig. 3:**
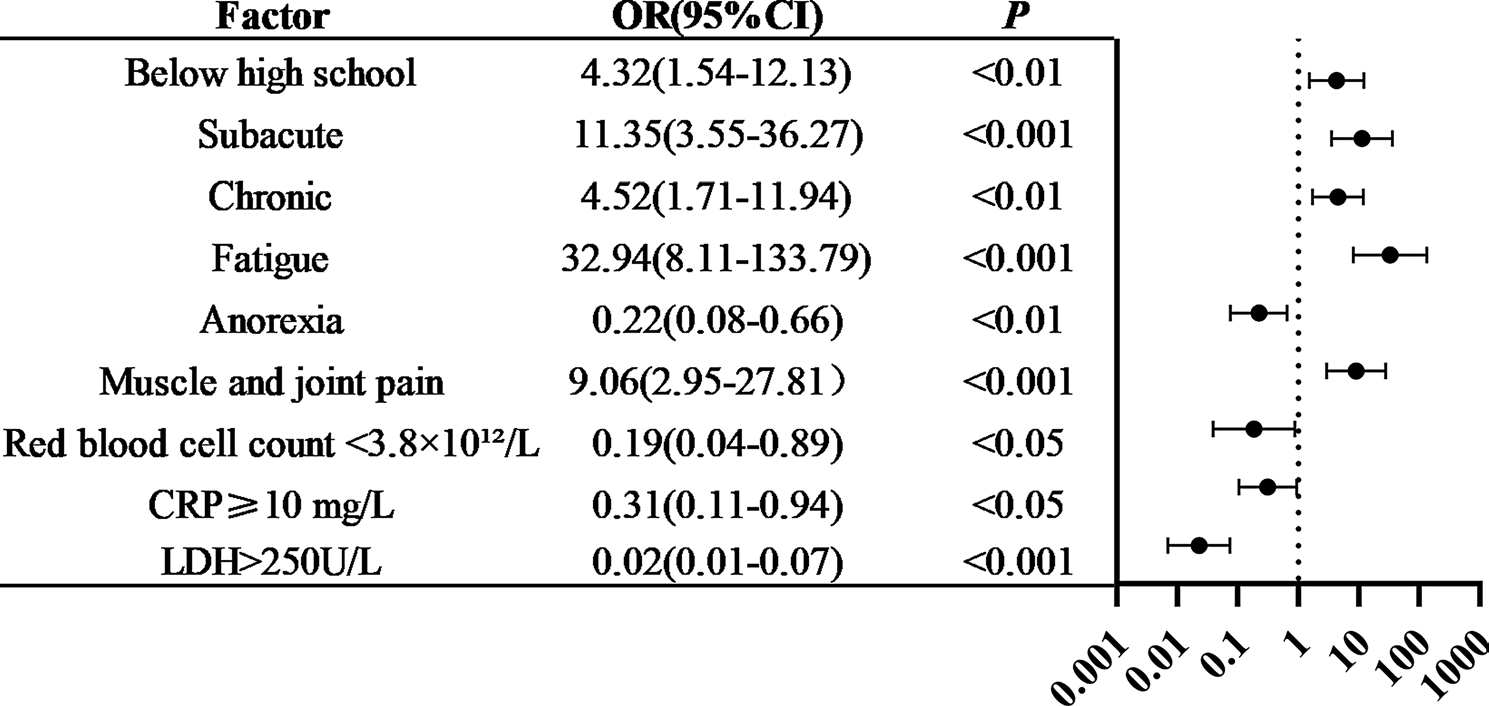
Forest plot of risk factors in the BrSpA group compared with the non-osteoarthritis brucellosis group

Independent risk factors for the development of BrSpA (Fig. 3): below high school education (*P* < 0.01, OR = 4.3, 95%CI: 1.5–12.1), subacute stage (*P* < 0.001, OR = 11.4, 95%CI: 3.6–36.3), chronic stage (*P* < 0.01, OR = 4.5, 95%CI: 1.7–11.9), fatigue(*P* < 0.001, OR = 32.9, 95%CI: 8.1-133.8), anorexia (*P* < 0.01, OR = 0.2, 95%CI: 0.1–0.7), muscle and joint pain (*P* < 0.001, OR = 9.1, 95%CI: 3.0-27.8), red blood cell count < 3.8 × 10^12^/L (*P* < 0.05, OR = 0.2, 95%CI: 0.0-0.9), CRP≥10 mg/L (*P* < 0.05, OR = 0.3, 95%CI: 0.1–0.9), and LDH > 250U/L (*P* < 0.001, OR = 0.0, 95% CI: 0.0-0.1).

After adjusting for confounding factors, multivariable logistic regression analysis identified independent factors associated with BrSpA. Collinearity diagnosis was performed for covariates included in the final multivariable logistic regression model. The maximum variance inflation factor (VIF) was 1.57, well below the conventional threshold of 10, indicating no obvious multicollinearity. The Omnibus test (*p* < 0.001) supported the overall statistical significance of the final model. The Hosmer–Lemeshow test yielded *p* = 0.531, indicating satisfactory model fitting. The Nagelkerke R² was 0.794, suggesting acceptable explanatory capability of the model.

To further explore the potential confounding effects of disease stage and socioeconomic status, stratified analyses were conducted according to disease stage (acute, subacute, chronic) and educational level (below high school vs. high school and above). As presented in Tables 4 and 5, the direction and strength of the main associations remained generally consistent across all subgroups, and the significant associations were preserved in most strata. These findings suggested that the observed associations were relatively robust and not substantially altered by disease stage or educational level.

**Table 4:** Disease stage–stratified analysis

Disease stage		B	OR	95% CI	*P*
**Acute**	Muscle and joint pain				
	No		1.00		
	Yes	2.78	16.09	2.79–92.65	0.002
	**Red blood cell count**				
	≥ 3.8 × 10¹²/L		1.00		
	< 3.8 × 10¹²/L	-2.24	0.11	0.01–0.80	0.03
	**LDH**				
	≤ 250 U/L		1.00		
	> 250 U/L	-3.14	0.04	0.01–0.20	< 0.001
**Subacute**	**Muscle and joint pain**				
	No		1.00		
	Yes	3.11	22.51	1.13-446.79	0.04
	**LDH**				
	≤ 250 U/L		1.00		
	> 250 U/L	-3.64	0.03	0.00-0.17	< 0.001
**Chronic**	**Education level**				
	High school and above		1.00		
	Below high school	3.58	35.99	5.15-251.65	< 0.001
	**Red blood cell count**				
	≥ 3.8 × 10¹²/L		1.00		
	< 3.8 × 10¹²/L	-2.79	0.06	0.01–0.54	0.012
	**Muscle and joint pain**				
	No		1.00		
	Yes	4.12	61.69	2.28-1668.53	0.014
	**CRP**				
	< 10 mg/L		1.00		
	≥ 10 mg/L	-3.32	0.04	0.00-0.48	0.012
	**LDH**				
	≤ 250 U/L		1.00		
	> 250 U/L	-4.95	0.01	0.00-0.10	< 0.001

**Table 5:** Education level–stratified analysis

Education level		B	OR	95%CI	*P*
**Below high school**	**Disease stage**				
	Acute		1.00		
	Subacute	2.39	10.91	3.58–33.31	< 0.001
	Chronic	2.01	7.44	2.71–20.42	< 0.001
	**Fatigue**				
	No		1.00		
	Yes	2.65	14.1	4.05–49.13	< 0.001
	**Anorexia**				
	No		1.00		
	Yes	-1.14	0.32	0.11–0.92	0.022
	**Muscle and joint pain**				
	No		1.00		
	Yes	2.13	8.43	2.94–24.21	< 0.001
	**Red blood cell count**				
	≥ 3.8 × 10¹²/L		1.00		
	< 3.8 × 10¹²/L	-1.54	0.21	0.07–0.66	0.022
	**LDH**				
	≤ 250 U/L		1.00		
	> 250 U/L	-3.55	0.03	0.01–0.09	< 0.001
**High school and above**	**Disease stage**				
	Acute		1.00		
	Subacute	2.35	10.45	1.50–72.60	0.018
	**Fatigue**				
	No		1.00		
	Yes	2.93	18.68	1.50-232.40	0.023
	**Anorexia**				
	No		1.00		
	Yes	-2.97	0.05	0.00-0.61	0.019
	**Muscle and joint pain**				
	No		1.00		
	Yes	3.84	46.28	4.65-460.89	0.001
	**Red blood cell count**				
	≥ 3.8 × 10¹²/L		1.00		
	< 3.8 × 10¹²/L	-2.3	0.05	0.00-0.71	0.027

### Univariate analysis of risk factors: BrSpA vs. healthy control group

The Univariate analysis of risk factors showed (Table 6) that the percentage of male patients, below high school education, farming and pastoral areas residence, farming and pastoral-related occupations were higher in patients with BrSpA than in healthy controls (all *P* < 0.05). The incidence of fever, hyperhidrosis, fatigue, anorexia, splenomegaly, and muscle and joint pain symptoms was higher in patients with BrSpA than in the healthy control group (*P* < 0.05). The incidence of decreased red blood cell count, elevated ESR, CRP, and LDH was higher in patients with BrSpA than in the healthy control group (*P* < 0.05). The difference between the two groups of patients in the remaining indicators was not statistically significant (all *P* > 0.05).

**Table 6:** Univariate analysis of risk factors: BrSpA vs. healthy control group

Factor	BrSpA group (*n* = 187)	Healthy control group (*n* = 279)	*P*
**Gender**			
Male	138(73.8%)	127(45.5%)	< 0.001
Female	49(26.2%)	152(54.5%)	
**Age**			
P50 (P25, P75)	51(41,60)	52(49,66)	0.135
**Education level**			
Below high school	158(84.5%)	137(49.1%)	< 0.001
High school and above	29(15.5%)	142(50.9%)	
**Region**			
Farming and pastoral areas	165(88.2%)	104(37.3%)	< 0.001
Non-farming and pastoral areas	22(11.8%)	175(62.7%)	
**Occupation**			
Farming and pastoral-related occupations	132(70.6%)	71(25.4%)	< 0.001
Other occupations	55(29.4%)	208(74.6%)	
**Fever**			
Yes	71(38.0%)	14(5.0%)	< 0.001
No	116(62.0%)	265(95.0%)	
**Hyperhidrosis**			
Yes	57(30.5%)	11(3.9%)	< 0.001
No	130(69.5%)	268(96.1%)	
**Fatigue**			
Yes	95(50.8%)	19(6.8%)	< 0.001
No	92(49.2%)	260(93.2%)	
**Anorexia**			
Yes	69(36.9%)	14(5.0%)	< 0.001
No	118(63.1%)	265(95.0%)	
**Muscle and joint pain**			
Yes	179(95.7%)	26(9.3%)	< 0.001
No	8(4.3%)	253(90.7%)	
**Lymphadenopathy**			
Yes	7(3.7%)	9(3.2%)	0.764
No	180(96.3%)	270(96.8%)	
**Splenomegaly**			
Yes	16(8.6%)	6(2.2%)	0.001
No	171(91.4%)	273(97.8%)	
**White blood cell count**			
< 3.5 × 10⁹/L	1(0.5%)	7(2.5%)	0.215
**Lymphocyte count**			
< 1.1 × 10⁹/L	14(7.5%)	29(10.4%)	0.288
**Neutrophil count**			
< 1.8 × 10⁹/L	6(3.2%)	10(3.6%)	0.827
**Red blood cell count**			
< 3.8 × 10¹²/L	15(8.0%)	2(0.7%)	< 0.001
**Hemoglobin level**			
< 110 g/L	21(11.2%)	17(6.1%)	0.047
**Platelet count**			
< 100 × 10⁹/L	1(0.5%)	1(0.4%)	0.777
**ESR**			
≥ 15 mm/h	160(85.6%)	57(20.4%)	< 0.001
**CRP**			
≥ 10 mg/L	144(77.0%)	9(3.2%)	< 0.001
**LDH**			
> 250 U/L	8(4.3%)	2(0.7%)	0.009
**AST**			
> 40 U/L	39(20.9%)	41(14.7%)	0.084
**ALT**			
> 40 U/L	32(17.1%)	35(12.5%)	0.168

### Multifactorial logistic analysis of risk factors: BrSpA vs. healthy control group

Relevant factors that were statistically significant in univariate analysis were used as independent variables, and variables with *P* values < 0.05 were included in the multivariate logistic regression model for analysis. The results of the multivariate analysis showed that residence in agricultural and pastoral areas, fever, anorexia, muscle and joint pain, ESR ≥ 15 mm/L, and CRP ≥ 10 mg/L are independent risk factors for the development of brucellar osteoarthritis in healthy individuals (*P* < 0.05). Detailed information is presented in Table 7.

**Table 7:** Multifactorial logistic analysis of risk factors: BrSpA vs. healthy control group

Factor	B	OR	95%CI	*P*
**Region**				
Non-farming and pastoral areas		1.00		
Farming and pastoral areas	2.11	8.21	1.65–40.75	0.01
**Fever**				
No		1.00		
Yes	3.78	43.92	3.98-484.68	< 0.01
**Anorexia**				
No		1.00		
Yes	3.84	46.34	2.98-720.73	< 0.01
**Muscle and joint pain**				
No		1.00		
Yes	5.71	302.54	44.04-2078.46	< 0.001
**ESR**				
< 15 mm/h		1.00		
≥ 15 mm/h	3.30	27.08	5.65-129.77	< 0.001
**CRP**				
< 10 mg/L		1.00		
≥ 10 mg/L	2.81	16.63	2.64-104.91	< 0.01

**Fig. 4:**
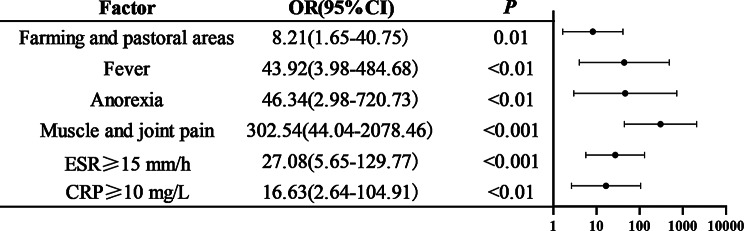
Forest plot of risk factors in the BrSpA group compared with healthy controls

Independent risk factors for the development of BrSpA in healthy individuals (Fig. 4): residence in farming and pastoral areas (*P* = 0.01, OR = 8.2, 95%CI: 1.7–40.8), fever (*P* < 0.01, OR = 43.9, 95%CI: 4.0-484.7), anorexia (*P* < 0.01, OR = 46.3, 95% CI: 3.0-720.7), muscle and joint pain (*P* < 0.001, OR = 302.5, 95%CI: 44.0-2078.5), ESR ≥ 15 mm/L (*P* < 0.001, OR = 27.1, 95%CI: 5.7-129.8), CRP ≥ 10 mg/L (*P* < 0.01, OR = 16.6, 95% CI: 2.6-104.9).

## Discussion

Our research has achieved a series of significant results. Initially, when comparing patients with BrSpA to those with non-osteoarticular brucellosis, we found that the proportion of BrSpA patients with an educational level below high school, residing in farming regions, engaging in farming occupations, having an abnormal BMI, and being in the subacute or chronic stages of the disease was significantly higher.In terms of clinical symptoms, compared to the non-osteoarticular brucellosis patient group, BrSpA patients had a lower proportion of cases with fever, hyperhidrosis, and anorexia, but a higher proportion of cases with fatigue and musculoskeletal pain.Regarding blood tests, compared to the non-osteoarticular brucellosis group, the BrSpA group had a lower incidence of decreased counts of white blood cells, lymphocytes, neutrophils, red blood cells, hemoglobin, and platelets, as well as a lower incidence of elevated levels of CRP, LDH, AST, and ALT.

When comparing BrSpA with non-osteoarticular brucellosis, several independent risk factors were identified, including having an educational level below high school, being in the subacute or chronic stage of the disease, experiencing fatigue, and suffering from muscle and joint pain. Interestingly, our study found a strong correlation between anorexia, decreased erythrocyte count, elevated CRP levels, and elevated LDH levels and the onset of BrSpA. The observed inverse associations between these factors and BrSpA are more likely to be explained by differences in disease stage rather than true biological protection. Traditionally, it is well - known that patients with BrSpA are often considered to have more severe manifestations compared to those with non - osteoarticular brucellosis. Consistent with previous studies, the probability of bone and joint involvement is higher in the subacute and chronic stages of brucellosis than in the acute stage [16, 22]. As the disease progresses gradually from the acute stage to the chronic stage, patients transition from having systemic acute symptoms to experiencing focal target organ damage. During this transition, the patient’s systemic acute symptoms may decrease. Meanwhile, the likelihood of target organ damage increases. This phenomenon is not unique to brucellosis and has also been demonstrated in other chronic diseases [23, 24].

To validate our hypothesis, we collected additional data from over 270 healthy controls. We then performed univariate and multivariate logistic analyses with the BrSpA group.When comparing BrSpA patients to healthy controls, we found that the former had significantly higher proportions of males, individuals with an educational level below high school, residents of farming regions, and those engaged in farming occupations. Clinically, BrSpA patients demonstrated elevated incidences of fever, hyperhidrosis, fatigue, anorexia, splenomegaly, and musculoskeletal pain. Hematologically and biochemically, they presented with higher rates of decreased red blood cell counts, ESR and CRP levels, as well as increased LDH compared to the control group.

In line with our hypothesis, in comparison to patients with non-osteoarticular brucellosis, anorexia acts as a “negatively correlated factors” against BrSpA. However, for healthy individuals, anorexia serves as a risk factor for BrSpA. The same pattern is observed with elevated CRP levels. During the univariate analysis phase, comparisons between the BrSpA group and the healthy control group revealed that both decreased red blood cell count and increased LDH levels had *P*-values < 0.001, suggesting a statistical association between these two indicators and BrSpA at the univariate level. Nevertheless, in the subsequent multivariate binary logistic regression analysis, after accounting for other potential confounding factors, neither decreased red blood cell count nor increased LDH levels were retained in the final model, indicating that they may not be independent influencing factors for the outcome variable.

The organs of the reticuloendothelial system have a high affinity for Brucella, and when it invades the liver, it causes changes in liver enzymes such as AST and ALT [25, 26]. The liver is an important part of the digestive system and is responsible for secreting bile and metabolizing nutrients. The impaired liver function affects digestive function and leads to symptoms such as anorexia, nausea and vomiting. Turan Buzgan et al. [27] have found that the elevation of AST occurs most often in the acute stage, while the changes in the chronic stage are not significant.

Particular attention should be paid to the fact that when CRP decreases to normal in patients with brucellosis, it does not necessarily mean that the disease is cured, but rather that there is a concomitant possibility of entering the chronic phase, resulting in damage to target organs such as bones and joints. CRP is one of the markers that can reflect the level of inflammation in the body to a certain extent [22]. It has been reported in many studies that elevated CRP may be associated with brucellosis [28–31], that it is highly susceptible to brucella infection, and that it may be used to determine the acute stage of brucellosis [32–35]. Thus, in our study, elevated CRP turned out to be a protective factor when BrSpA group was compared with the non-osteoarticular brucellosis group. This may be since in patients with BrSpA, the disease course is in the middle to late stages, and the immune system has gradually adapted, resulting in a weakening of the inflammatory response and a decrease in CRP to normal.

Studies have indicated that individuals with higher educational attainment exhibit greater awareness of infectious diseases such as tuberculosis, which can, to a certain extent, reduce disease transmission. They are more likely to seek medical attention promptly upon experiencing symptoms, thereby minimizing the occurrence of complications and demonstrating higher treatment adherence during the course of illness [36]. As educational levels rise, the incidence of brucella infection in the population also shows a gradual decline [37]. The findings of the present study align with this perspective, revealing that patients with an educational level below high school are at a 4.3-fold increased risk of developing BrSpA following brucella infection compared to those with a high school education or higher.

The subacute and chronic stage are independent risk factors for BrSpA in brucellosis patients with OR values of 11.4 and 4.5, respectively. This is supported by the literature, where a higher probability of bone and joint involvement was observed in subacute versus chronic phase compared to acute phase [16].

Although both fatigue and anorexia are systemic manifestations, it is noteworthy that the chronic infection phase of brucella is mainly characterized by chronic fatigue-like syndrome-like manifestations such as fatigue [38, 39]. When entering the stage of target organ damage, the probability of worsening fatigue is higher, although patients’ symptoms of anorexia improve.

Muscle and joint pain were an independent risk factor for the development of brucellosis to BrSpA, with an OR of 9.1. There is no doubt that the risk of muscle and joint pain is exacerbated by damage to target organs such as bones and joints after entering the middle to late stages of the disease.

In this study, a total of 363 patients with brucellosis were included, among whom the proportion of patients with BrSpA reached as high as 51.5%. This finding is largely consistent with the 51% prevalence rate reported by Lei Zhu et al. [40] in their 2024 study. It suggests that the incidence of BrSpA remains at a relatively high level, posing an ongoing challenge that demands urgent attention. Among the 187 patients with BrSpA, spondylitis accounted for the highest proportion, with lumbar spine involvement being particularly prominent, which is in line with the findings reported by Esmaeilnejad-Ganji et al. [5]. Exploring the risk factors for BrSpA is significant for preventing musculoskeletal complications in patients with brucellosis.

Consistent with previous studies, our findings indicate that among patients with brucellosis, males outnumber females, a trend also reported by Dean, A. S. et al. [41]. This discrepancy may be attributed to differences in work. The incidence of human brucellosis primarily hinges on the frequency of contact with livestock, thus giving the disease a distinct occupational predisposition [42]. Brucellosis is prevalent in agriculture and livestock related work, and men are more likely to be engaged in veterinary work than women in occupations such as being breeders, slaughterers and those engaged in fur processing, which have frequent contact with domestic animals and their products. Therefore, appropriate health education and occupational exposure prevention for people working in agro-pastoral related jobs is essential to reduce the incidence of brucellosis.

Certain limitations of this study should be acknowledged. Although this research was based on multicenter retrospective case analysis, the overall sample size was still relatively limited. A smaller sample may introduce sparse data bias, model overfitting, and inflated effect size estimates. In addition, potential confounding factors related to disease stage and socioeconomic status could not be fully adjusted in the current analysis, which may weaken the robustness of the observed associations. The diagnosis of BrSpA relied primarily on routine clinical judgment and imaging findings without unified standardized confirmatory criteria, which may affect diagnostic validity. Future studies should adopt standardized diagnostic protocols to improve consistency and accuracy. Furthermore, given the inherent multicenter retrospective design, complete blinding was difficult to achieve. Although we minimized potential bias by implementing blinding procedures at key assessment and analytical stages, the possibility of case misclassification could not be entirely ruled out. Accordingly, the clinical applicability and generalizability of our findings remain to be verified in external independent cohorts. Large-sample, multicenter prospective studies with long-term follow-up are warranted to overcome the above limitations and confirm our results.

## Conclusion

The atypical clinical course of brucellosis makes early identification of its progression to BrSpA a major clinical challenge. Our findings are consistent with the hypothesis that clinicians should maintain a high index of suspicion when patients progress to the subacute or chronic stage and present with a paradoxical clinical pattern: resolution of systemic inflammatory symptoms, accompanied by improved appetite and normalized red blood cell count, LDH and CRP levels. This distinct clinical profile may be associated with an elevated risk of osteoarticular complications, prompting clinicians to reassess the risk of disease progression and consider individualized adjustments to treatment strategies. In contrast, patients without this paradoxical profile are more likely to remain in the acute stage, for whom timely antimicrobial therapy remains the priority to prevent subsequent osteoarticular involvement. Importantly, due to the inherent limitations of the retrospective case–control design, no causal inference can be drawn from the present findings. The observed associations need further validation in large‑scale, multicenter prospective cohorts with prolonged follow-up before they can be established as reliable clinical warning indicators. 

## Data Availability

The data presented in this study are available on request from the corresponding author.

## Abbreviations

BrSpA: Brucellar Spondyloarthritis
OR: Odds ratio
CRP: C-reactive protein
FAO: Food and Agriculture Organization of the United Nations
WHO: World Health Organization
SpA: Spondyloarthritis
NSAIDs: Non-Steroidal Anti-Inflammatory Drugs
ESR: Erythrocyte Sedimentation Rate
LDH: Lactate Dehydrogenase
AST: Aspartate aminotransferase
ALT: Alanine aminotransferase
RBPT: Positive Rose Bengal Plate Test
SAT: Standard Agglutination Test
CT: Computed Tomography
MRI: Magnetic Resonance Imaging
CI: Confidence intervals
